# EBF1-mediated up-regulation of lncRNA FGD5-AS1 facilitates osteosarcoma progression by regulating miR-124-3p/G3BP2 axis as a ceRNA

**DOI:** 10.1186/s13018-022-03181-7

**Published:** 2022-06-27

**Authors:** Ou Shuang, Jianmin Zhou, Zijun Cai, Longteng Liao, Yuehua Wang, Wenyu Wang, Meng Xu

**Affiliations:** 1 Department of Orthopedics, Shangrao People’s Hospital, No. 86, Shuyuan Road, Shangrao, 334000 Jiangxi China; 2 Orthopaedic Medical Center, the Second Hospital of Jilin University, No. 218, Ziqiang Street, Changchun, 130041 Jilin China

**Keywords:** FGA5-AS1, miR-124-3p, G3BP2, Osteosarcoma

## Abstract

**Background:**

As a skeletal malignancy, osteosarcoma has high incidence among primary malignant bone tumors. With increasing researches on molecules which mediate cancer progression, molecular mechanism has gradually become the pivot of osteosarcoma research and treatment.

**Aim:**

Our study aimed at investigating the function of G3BP stress granule assembly factor 2 (G3BP2), which is an oncogene for breast cancer (BC) and prostate cancer but remains unknown in osteosarcoma cells.

**Methods:**

Related gene expression was confirmed by RT-qPCR. Functional assays including immunofluorescence (IF), colony formation, transferase-mediated dUTP nick-end labeling (TUNEL) as well as transwell assays were utilized to test the cell biological process caused by the genes. Meanwhile, RNA pull-down assay, along with luciferase reporter and RNA immunoprecipitation (RIP) assays, was utilized to detect the interaction G3BP2, miR-124-3p and FGD5 antisense RNA 1 (FGD5-AS1) may exert on the regulation of osteosarcoma cells.

**Results:**

G3BP2 was with high expression in osteosarcoma cells, and it aggravated the malignant cell behaviors in osteosarcoma. Additionally, miR-124-3p was verified to negatively regulate G3BP2 expression in osteosarcoma cells. Moreover, lncRNA FGD5-AS1 was predicted and testified to be the sponge of miR-124-3p and modulated G3BP2 expression positively. Subsequently, FGA5-AS1 accelerated osteosarcoma cell proliferation through up-regulating G3BP2. Furthermore, we identified EBF transcription factor 1 (EBF1) as the transcription factor for FGA5-AS1, and EBF1 served as a tumor facilitator in osteosarcoma cells.

**Conclusion:**

EBF1 induced-FGA5-AS1 aggravated osteosarcoma cell malignancy by targeting miR-124-3p and G3BP2.

**Supplementary Information:**

The online version contains supplementary material available at 10.1186/s13018-022-03181-7.

## Background

As a skeletal malignancy, osteosarcoma has high incidence among primary malignant bone tumors. It primarily occurs among children and young people [1]. At present, the common treatment methods including wide tumor excision, neoadjuvant or adjuvant chemotherapy and radiotherapy have helped to increase the 5-year survival of the non-metastatic patients to 65% [2]. The extreme variability of osteosarcoma makes the combined therapies rather than a single target therapy more important and necessary in the treatment of osteosarcoma patients [3]. Having an understating on the molecular mechanism and exploration of effective therapeutic options for osteosarcoma are thus full of significance.

As a member of the G3BP family, G3BP stress granule assembly factor 2 (G3BP2) has been reported to be highly expressed in cancer cells and exert carcinogenic influence in certain cancers by different researches. For instance, G3BP2 could induce prostate cancer cell cycle progression and accelerate tumor growth, while block cell apoptosis [4]. G3BP2 could also regulate breast tumor initiation [5]. However, its functions on osteosarcoma are still unclear.

Long non-coding RNAs (lncRNAs) are defined as a member of non-coding RNA (ncRNA) family, comprising more than 200 nucleotides in length without the ability to code proteins [6]. LncRNAs have been confirmed to take part in cancer cell biological processes, such as cell proliferation, migration and apoptosis [7]. For example, lncRNA RMRP was reported to accelerate the cell malignancy in bladder cancer via miR-206 [8]. In recent years, lncRNA FGD5 antisense RNA 1 (FGD5-AS1) has been proven to exert carcinogenic influence in different cancer types. For instance, FGD5-AS1 promoted cell proliferative capability of colorectal cancer (CRC) via sponging miR-302e to up-regulate CDCA7 [9]. Besides, FGD5-AS1 could accelerate non-small cell lung cancer (NSCLC) cell proliferative capability by regulating miR-107/FGFRL1 axis [10]. Furthermore, it was also proven that FGD5-AS1 promoted tumor growth of oral cancer by sponging miR-153-3p and regulating MCL1 [11]. The functions of lncRNAs on osteosarcoma were also studied. For instance, lncRNA TTN-AS1 regulated osteosarcoma cell apoptosis as well as drug resistance through the miR-134-5p/MBTD1 pathway [12]; lncRNA SNHG4 sponged miR-377-3p to aggravate osteosarcoma cell proliferation as well as migration [13], and lncRNA BE503655 inhibited the osteosarcoma cell malignancy via Wnt/beta-catenin pathway [14].

As an evolutionarily conserved single-stranded RNA comprising 21–24 nucleotides [15], microRNA (miRNA) exerts crucial functions in the post-transcriptional regulation of messenger RNAs (mRNAs) through targeting mRNA 3’ UTR, resulting in the translational inhibition of mRNAs [15]. For example, miR-491 could repress cell migration of osteosarcoma via targeting αB-crystallin [16]. MiR-486 inhibited EMT process of osteosarcoma by targeting PIM1 [17].

In this research, our main purpose is to display the biological functions and potential regulatory mechanism of G3BP2 in osteosarcoma. Moreover, the interaction between G3BP2 and FGD5-AS1 and other RNAs were explored by a series of mechanism assays, which might offer a new insight for curing osteosarcoma.

## Methods

### Cell culture

We obtained 1.5 × 10^5^ osteosarcoma cell lines (Saos-2, U2OS, MG-63 and HOS) and the normal osteoblast cell line HFOB1.19 from ATCC (Manassas, VA, USA). McCoy’s 5a medium (16600082, GIBCO, Rockville, MD, USA) was utilized to cultivate U2OS and Saos-2 cells. MG-63 and HOS cells were cultivated in Eagle’s Minimum Essential Medium (21010046, GIBCO). HFOB1.19 was cultivated in DMEM-F12 medium (11320033, GIBCO). Next, 10% fetal bovine serum (FBS; 10100147, GIBCO) served as the supplement in culture medium. All cells mentioned above were deposited at 37 °C with 5% CO_2_, except HFOB1.19, which was deposited at 33.5 °C with 5% CO_2_.

### Cell transfection

The specific short hairpin RNAs (shRNAs) targeting G3BP2, FGD5-AS1 and EBF1 as well as their negative control (NC) plasmids were structured through GeneChem (Shanghai, China). Also, NC mimics/inhibitor and miR-124-3p mimics/inhibitor were purchased from RiboBio (Guangzhou, China). Besides, pcDNA3.1/G3BP2 and pcDNA3.1 NC vectors were accordingly implemented by GeneChem. Cell transfection was performed with Lipofectamine 3000 (Invitrogen) for 48 h. Bio-repeats run in triplicate.

### RT-qPCR

Firstly, we obtained the total RNA from cells by TRIzol reagent (Invitrogen). Then, the total RNA was reversely transcribed into cDNA by PrimeScript™ RT reagent kit (TaKaRa, Shiga, Japan). Next, based on the user guide, qPCR was conducted through SYBR green Supermix (Thermo Fisher, Waltham, MA, USA). Finally, the gene expression level was measured via 2^−ΔΔCt^ method. Moreover, GAPDH and U6 were internal controls. Bio-repeats run in triplicate. The sequences used are listed in Table 1 for reference.

**Table 1:** The primers’ sequences which were taken for RT-qPCR have been listed

Primers	Sequences (5' > 3')
G3BP2	taaggcacgcggtgaatgccaag
miR-25-3p	cattgcacttgtctcggtc tga
miR-92a-3p	tattg cacttgtccc ggtctt
miR-367-3p	aattgca ctttagcaat ggtga
miR-92b-3p	tattgcactc gtcccggcct cc
miR-32-5p	tattg cacattacta agttgca
miR-363-3p	a attgcacggtatccatctgta
miR-124-3p	taaggcacgcggtgaatgccaag
miR-101-3p	tacagtactgtgataactga
FGD5-AS1	aattagccagcccatccgtcgctcacgaggtcgcgcctcgcacccgccttcctccttttcttttaccctccccttcagaaaaaacggcggttgggctgcgacggccgccaaaggcggactagaagcggaggggtgaaaatcccggcagagaaggaagaagggactgcaggcgggaggaggaggaggataaggaggaagggagcccgcccagccggagccatctccgtcgagaacaaaatggcggcgctggcggagggccaactataaggcggggccgcggccattccccctccacccccacccttgcgccggccgggccggtcagggggaacccgctcgcttcgcccggccgcgggcggggaggggaggggagcggcccggcccactatgcaaagcgccgggcgccgccgccgccaccccgtggcaaagaatatgctttttgtctaatgatgttagataaaaagcaaatttggagcagttttcttattcgagttcaaaatggttcataaagcagcgaagacaactcaaaacatcagtaacacatttggcccaggaactgctaacaaacatacagtgcagtggtggcttaagaagttttgcaaggaagagagccttgaagatgaggaacgtgatatgggccatgggaagttgacaacgaccaattgagagcaatcattgaactacatgaaaaattacagaagaactcagcgttgactattctacggtcattagggtcgttcagcattcgaagcaaattggaaaggtgaaaaagcttgataagtgggtgcctcatgagctgagcggaaatcaaaactatcgttttgaagtgtagtcttcttttatgctacgcaacaacaaaccatttctcaatcggattgtgacatgtaatgaaaagtgaattttatacaacaaccagctcagtggttggaccaagaggcagctccaaagcagttcccaaagccaaacttgcaccaaaaaaaaggtcatggtcactgttcggtggtctgctgcccatctgatccactacagttttctgaatcctggtgaaaccattacatctgagaagtatgctcagcaaattggtaagatgcaccgaaaactgcaatacctgctgcctgcattggtcaacagaaagggcccaattcttctccacgacaacacctgactgcgtgtcgcacaaccagtgcttcaaaagttgaaggaattgggctacaaagttttgcctcatccaccatattcacctgacctttcgccaactgactaccacttctgctagcatcttgacaactttttgcagggaaaacacttcagcatggtgcagaaaatgctttccaagagttcgtcaaatcctgaagcacggatttttatgctacaggaataaacaaacatttctcattggcaaaaatgtgttgattgtaatggttcctgttttgattaatagaatatgttttagcgtagttacaatgatttaaaattcgcagtctggaaatttaatttttgcatcaacctaatatttctatggtaaatccttgcaaacatggaaacaatgcatttggcccagtgctttgtggttgtgtactctttttctttgtttttttaatagatggcattggccgggcatggtggctcatgcctgtaatcccagcattttgggaaggtgaggtgggtggatcacctgaggtcaggagttcaagaccagcctgactaacatggtaaaaccccatctctactaaaaatacaaaaaaattagctaggcggggtggcgggcatctaattccagctacttcatgaggctgcgacaggagaatcatttgaactcgggaggcagaggttgcagtgagccgagatcacaccattgcactccagcctgggcgatgagcgaaactgtctcaaaaaaaaaaaaagttggggtctcgctgtgttgaccaggctggtctcgaactcctggcctcaagtgatcctcccatcttggcctcccaaagtgttaggattacaggcgtgagccaccacaccctgcctggttgtgtactcttttaaatactaagtttttaatgttaaatgctgcttttagatacactgtaaaaatacacctatcaatgagtttttttattaaaaacattgcaattgtactagactttaaatactaagcaataattcaggcttcaatgttggtttatagttttctcatttctttcatttaatacctctgtaaaatgaagcagttacttccattttcctgaggtgagataagtgccctgcacaatgttataggcccagtaagtgagactggagctctgatctaatccctttgacattaaagtttgtgcagtaaaccagctatgtcagtttgccacatttgtaatagtgttcaataaattttcacttaaaggtttaatattaaagtagtagcaagaattcataaccttaattggctttaacagcttttctttccctgaaaacagatgcatatgtacacatgcacacagattgcttacaatatcagcacgtaaaaggcctttgcacattgaagtcggcactgctttggtgccttttttgttttttggctcggtgttttgactgcaagtctttttggatagaattttatagttagaaagtagctaacacttgggttttataggcacaaaaaacaagtcttatactagctgtactttattttttgagttcttattaatgaggaacatccacttttgcattgacagtgatttcaagattgctttatcagcctttaaaggattcttgactagtcgtgcacatcagaactgccaggtccccagtggttctgaagcagtaagctttgggtgggctctggcatcagcactttcactaagcttcacagataattctgatgcatactccaggcctgaaccactgatcaatttgaaacatgcataacaaagcaaaaaaagttttgtttcaccttttgaaatacagttaactcttttaccatgccagagatcattcagagagacaggtcgttgctccggagtgatacagatctggcagtacccagcccttgtgtgtgtgcgttagctcagcacctgcccacactgcgagcccccgtaggatgtgccttgtccttccctgtttcagcacttaacacactacctggtacagagtatgtagtgggcatctgttgaatgaatgcttttcccagtagcagtgtattcatacaatattaatataattgtcccctggcttacagataaaaatgaaagcatcaagtgcccagtgagtgagacccaggtgttcttcctccacccctagtggtcccctgggcaggtctttttttttttgtaacactcaccagtctgttctgtagtcaatcattgattgacttgtctgtgaacttggaggaactgtttcatagtttcattagcacagagtaaacatgtttgccatgcaaggttattttgcatctgcatttaagtgataatgttgaatcaatgaaaagtgttgattaagcagtagttgtagatatgctaagtttttcaaattactaatatcaagtggagattgtttttacttttaagggtattgcttttgtgatagcataaataatggttttccttttttgtaatgtaaattaattgctggcaacttttgtattcccatagactggggaagcttaattgcctttacaagtacttatgtacaactttgtatcaaattttctgtaatagtttatgctttagtactatatatgtactaataattttatctgacttctgtttatatcatttgtacaattacatggttgtaaaataaacttttaaacctcacgtaa
NEAT1	ggagttagcgacagggagggatgcgcgcctgggtgtagttgtgggggaggaagtggctagctcagggcttcaggggacagacagggagagatgactgagttagatgagacgagggggcgggctgggggtgcgagaaggaagcttggcaaggagactaggtctagggggaccacagtggggcaggctgcatggaaaatatccgcagggtcccccaggcagaacagccacgctccaggccaggctgtccctactgcctggtggagggggaacttgacctctgggagggcgccgctcttgcatagctgagcgagcccgggtgcgctggtctgtgtggaaggaggaaggcagggagaggtagaaggggtggaggagtcaggaggaataggccgcagcagccctggaaatgatcaggaaggcaggcagtgggtgcagggctgcaggagggccgggagggctaatcttcaacttgtccatgccagcagcccctttttttccagaccaagggctgtgaacccgcctggggatgaggcctggtcttgtggaactgaacttagctcgacggggctgaccgctctggcccagggtggtatgtaattttcgctcggcctgggacggggcccaggccgggcccagcctggtggagcgtccaggtctgggtgcgaagccaggcccctgggcggaggtgaggggtggtctgaggagtgatgtggagttaaggcgccatcctcaccggtgactggtgcggcacctagcatgtttgacaggcggggactgcgaggcacgctgctcgggtgttggggacaacattgaccaacgctttattttccaggtggcagtgctccttttggacttttctctaggtttggcgctaaactcttcttgtgagctcactccaccccttcttcctccctttaacttatccattcacttaaaacattacctggtcatctggtaagcccgggacagtaagccgagtggctgttggagtcggtattgttggtaatggtggaggaagagaggccttcccgctgaggctggggtggggcggatcggtgttgcttgcctgcagagagggtggggagtgaatgtgcacccttgggtgggcctgcagccatccagctgaaagttacaaaaatgcttcatggaccgtggtttgttactatagtgttcctcatggcgagcagatggaaccgggagacatggagtccctggccagtgtgagtcctagcattgcaggaggggagaccctggaggagagagcccgcctcaattgatgcctgcagattgaatttccagaggcttaggaggaggaagttctccaatgttctgtttccaggccttgctcaggaagccctgtattcaggaggctaccatttaaagtttgcagatgagcttatggggggcaatcttaaaaagtccacagcagatgcatccggctcgaggggccatcagctttgaataaatgcttgttccagagcccatgaatgccagcaggcacccctcctttcctggggtaaaggttttcagatgctgcatcttctaaattgagcctccggtcatactagttttgtgcttggaaccttgcttcaagaagatccctaagctgtagaacattttaacgttgatgccacaacgcagattgatgccttgtagatggagcttgcagatggagccccgtgacctctcacctacccacctgtttgcctgccttcttgtgcgtttctcggagaagttcttagcctgatgaaataacttggggcgttgaagagctgtttaattttaaatgccttagactggggatatattagaggaagcagattgtcaaattaagggtgtcattgtgttgtgctaaacgctgggagggtacaagttggtcattcctaaatctgtgtgtgagaaatggcaggtctagtttgggcattgtgattgcattgcagattactaggagaagggaatggtgggtacaccggtagtgctcttttgttcttgcttcgtttttttaaacttgaactttacttcgttagatttcataatactttcttggcattctagtaagaggaccctgaggtgggagttgtgggggacggggagaaggggacagcttggcaccggtcccgtgggcgttgcagtgtgggggatgggggtatgcagcttggcactggtactgggagggatgagggtgaagaaggggagagggttggttagagatacagtgtgggtggtgggggtggtaggaaatgcaggttgaagggaattctctggggctttggggaatttagtgcgtgggtgagccaagaaaatactaattaataatagtaagttgttagtgttggttaagttgttgcttggaagtgagaagttgcttagaaactttccaaagtgcttagaactttaagtgcaaacagacaaactaacaaacaaaaattgttttgctttgctacaaggtggggaagactgaagaagtgttaactgaaaacaggtgacacagagtcaccagttttccgagaaccaaagggaggggtgtgtgatgccatctcacaggcaggggaaatgtctttaccagcttcctcctggtggccaagacagcctgtttcagagggttgttttgtttggggtgtgggtgttatcaagtgaattagtcacttgaaagatgggcgtcagacttgcatacgcagcagatcagcatccttcgctgccccttagcaacttaggtggttgatttgaaactgtgaaggtgtgattttttcaggagctggaagtcttagaaaagccttgtaaatgcctatattgtgggcttttaacgtatttaagggaccacttaagacgagattagatgggctcttctggatttgttcctcatttgtcacaggtgtcttgtgattgaaaatcatgagcgaagtgaaattgcattgaatttcaagggaatttagtatgtaaatcgtgccttagaaacacatctgttgtcttttctgtgtttggtcgatattaataatggcaaaatttttgcctatctagtatcttcaaattgtagtctttgtaacaaccaaataaccttttgtggtcactgtaaaattaatatttggtagacagaatccatgtacctttgctaaggttagaatgaataatttattgtatttttaatttgaatgtttgtgctttttaaatgagccaagactagaggggaaactatcacctaaaatcagtttggaaaacaagacctaaaaagggaaggggatggggattgtggggagagagtgggcgaggtgcctttactacatgtgtgatctgaaaaccctgcttggttctgagctgcgtctattgaattggtaaagtaataccaatggctttttatcatttccttcttccctttaagtttcacttgaaattttaaaaatcatggttatttttatcgttgggatctttctgtcttctgggttccattttttaaatgtttaaaaatatgttgacatggtagttcagttcttaaccaatgacttggggatgatgcaaacaattactgtcgttgggatttagagtgtattagtcacgcatgtatggggaagtagtctcgggtatgctgttgtgaaattgaaactgtaaaagtagatggttgaaagtactggtatgttgctctgtatggtaagaactaattctgttacgtcatgtacataattactaatcacttttcttcccctttacagcacaaataaagtttgagttctaaactcattagaaaaaaaaaaaaaaaaaaaaaaa
SNHG16	gcgttcttttcgaggtcggccgcgtggctggaagacatggccactccagtcggtgttgagcacggcgagcagtctcaggcctttagtgatgatgattaaaggtggtggctgtggccttgaaaacagtcatgtgaaaactcatcaccttaaggtgttaagtgtaaggatcttcatgatgaaatttctgtaaatggtgcagtcagcctcagtttccaaagccggaaaaggatcctctagtagccacggtgtggcagctgctctgaaccaggacctggacccggacccaaagtgccatgtctttaatgtgagttagctcccagcgatgccagatgggatcagcacagccctgcctctgctgctaattgttcctctaaagtaatcgccatgcgttctttgggcttcatctttaaaggaatgaagcaactgagattattctggaaaaccttttggcagttagtgaaattagagtacaactaagaacattttcagacctccactgtggatgacctgggtataatctcacaaatcgatgggactgcaaggattgtaaactgaaatgaacatgattatactctgttggaagagcctaagaggaaactgatgccatgagtttcagagagtaatgcttaaccccagttacacaggatgccgtcttgtgtttcctcttgtttagttacccactacagtgattttgtgatctgctaatgggttgccacccacaaccattgctttagcacttttacttcaaatcaatgaaggattgataaaagttctcctggtgtctccgcagagtgccttccaggaacagatctttgcatagaatatcagtggtttccttttttgtttcaaatagtggtcagaaaatacccagtgttgactcaccaaggcaatcagcttcctttttccctttttttgttttttttttaacattttatatttttgctttattttattttattttattttattttattttattttattttattttttgagacggagttccactctgtcgccaggctggagtgaagtggtacaatcttggctcactgcaacctccacctcccgggttcaagcaattctcctggctcagcctcctgagtgctgggactacaggcgcgtaccttctttagtagagactgggtttcaccatgttggccaggatggtctctatctcctgaccttgtgatctgcctgcctcagcttcccaaagtgctgagatgacaggtgtgagccatcagacccagcatttttttttttaatttaaatttaaattttttttcatttttttgagaggttttttttgttttgttttgttgttgttgttgttgttgtttttgagacagtcttgctctgtcacccaggctgggagtgcagtggcatgatctctgcaacctctacctcccaggttcaagcaattcttgtgcctcagcctcccaagtaactgggactacaggtgcacgctaccacacctggctgattttttttgttttagtagagacagggtttcaaccatgttgcccaggttggtctcaaactcctgagctcaggcaatccacccgccttggcctcccaaagtgctaggattacaggtgtgagccaccacacccagctattttttctttcgttttttaattttaaagttgggggggggtctcaatttgttaccctggctggtctcgaactcccggacttaagcgatcctctggctccaagcccactaccagtctcaggtttctttactaaaagatcactacctttttttctcttatctgctgccatgtgagatgtggctttcaccttccgccatgattgtgaggccttcccagccatgtagaactgtaagtccaataaacctcttttgtaaattaaaaaaaaaaaatcactatttaagatactaggatggattgtgactgttgaggagtacttacatatcctacatttgactacattatttccaaaccaagtattccatccaaaggaacatactgctatcatagagaccaaggagggactgtttaaggttgccaaggtgaagcgagctgagaggctttgtcctcgtgccagtaactctgaaatctctcttaattcctgctgtccaggcagcagaatgccatggtttccccaagtaggtagctgctttagcagttaaagcccaaatgtctgttctgttgatcagaggtctctgaatttctgaagtggtgtttcgtttctggtgactgagttaatcctttacaatccctcttgtaaagtgtgctaatagaaagaatccacctttcaaagctgcagaaccagaccgtgccctaaattgaccaacgtaactgatgtgcctcaggaagtctcttgccagctgtccctgtgaagacccccctcctcccccccagctgctgccttgcacactgaagcatctcagactgtgcaaagccgtgtagtcatcaagacagtaaatcccagggcttggttaagtgctgtgtgataacttgtttggatgagacttaacttaaaaccacttacaataaacttgggaaactaccgtca
GAPDH	gctctctgctcctcctgttcgacagtcagccgcatcttcttttgcgtcgccagccgagccacatcgctcagacaccatggggaaggtgaaggtcggagtcaacggatttggtcgtattgggcgcctggtcaccagggctgcttttaactctggtaaagtggatattgttgccatcaatgaccccttcattgacctcaactacatggtttacatgttccaatatgattccacccatggcaaattccatggcaccgtcaaggctgagaacgggaagcttgtcatcaatggaaatcccatcaccatcttccaggagcgagatccctccaaaatcaagtggggcgatgctggcgctgagtacgtcgtggagtccactggcgtcttcaccaccatggagaaggctggggctcatttgcaggggggagccaaaagggtcatcatctctgccccctctgctgatgcccccatgttcgtcatgggtgtgaaccatgagaagtatgacaacagcctcaagatcatcagcaatgcctcctgcaccaccaactgcttagcacccctggccaaggtcatccatgacaactttggtatcgtggaaggactcatgaccacagtccatgccatcactgccacccagaagactgtggatggcccctccgggaaactgtggcgtgatggccgcggggctctccagaacatcatccctgcctctactggcgctgccaaggctgtgggcaaggtcatccctgagctgaacgggaagctcactggcatggccttccgtgtccccactgccaacgtgtcagtggtggacctgacctgccgtctagaaaaacctgccaaatatgatgacatcaagaaggtggtgaagcaggcgtcggagggccccctcaagggcatcctgggctacactgagcaccaggtggtctcctctgacttcaacagcgacacccactcctccacctttgacgctggggctggcattgccctcaacgaccactttgtcaagctcatttcctggtatgacaacgaatttggctacagcaacagggtggtggacctcatggcccacatggcctccaaggagtaagacccctggaccaccagccccagcaagagcacaagaggaagagagagaccctcactgctggggagtccctgccacactcagtcccccaccacactgaatctcccctcctcacagttgccatgtagaccccttgaagaggggaggggcctagggagccgcaccttgtcatgtaccatcaataaagtaccctgtgctcaacca
U6	gtcccttcggggacatccgataaaattggaacgatacagagaagattagcatggcccctgcgcaaggatgacacgcacaaatcgagaaatggtccaaaatttt
EBF1	cctgcttcttcaagtgaagggtacctctacaaaaggaaactccagcccctcctgtcctccaccggcctgtgatcattacaaaaaaaaaaaaaaaaaagcaaaaaaaaaaaaaaagcacccaaaccaaaaatcaaccaaccaaacaacccccaacagccaagcatacatctctaattttattattttggtcttttcgttggattttccctttcttctttttttcgggttatcgctcagttttgagcagaggtttacattttttaaaaatttgctttccagcccgccttgatcttctaagtgcgagttcatcgtctgagaaaaaaaaaaatctctggttggcgtttttgtttcttttcttttctttcttttctttcctttttttttttttttaatttttttcaagggggaggagattttccacaagaaaaggttgttttcatgtttgggattcaggaaagcatccaacggagtggaagcagcatgaaggaagagccgctgggcagcggcatgaacgcggtgcggacgtggatgcagggcgccggggtgctggacgccaacacggcggcgcagagcggggtgggtctggcccgggctcactttgagaagcagccgccttccaatctgcggaaatccaacttcttccacttcgtcctggccctctacgacagacagggccagcccgtggagatcgagaggacagcgtttgtggggttcgtggagaaggaaaaagaagccaacagcgaaaagaccaataacggaattcactaccggcttcagcttctctacagcaatgggataaggacggagcaggatttctacgtgcgcctcattgactccatgacaaaacaagccatagtgtatgaaggccaagacaagaacccagaaatgtgccgagtcttgctcacacatgagatcatgtgcagccgctgttgtgacaagaaaagctgtggcaaccgaaatgagactccctcagatccagtgataattgacaggttcttcttgaaatttttcctcaaatgtaaccaaaattgcctaaagaatgcgggaaacccacgtgacatgcggagattccaggtcgtggtgtctacgacagtcaatgtggatggccatgtcctggcagtctctgataacatgtttgtccataataattccaagcatgggcggagggctcggaggcttgacccctcggaaggtacgccctcttatctggaacatgcagctactccctgtatcaaagccatcagcccgagtgaaggatggacgacgggaggtgcgactgtgatcatcataggggacaatttctttgatgggttacaggtcatattcggtaccatgctggtctggagtgagttgatcactcctcatgccatccgtgtgcagacccctcctcggcacatccctggtgttgtggaagtcacactgtcctacaaatctaagcagttctgcaaaggaacaccaggcagattcatttatacagcgctcaacgaacccaccatcgattatggtttccagaggttacagaaggtcattcctcggcaccctggtgaccctgagcgtttgccaaaggaagtaatactcaaaagggctgcggatctggtagaagcactgtatgggatgccacacaacaaccaggaaatcattctgaagagagcggccgacattgccgaggccctgtacagtgttccccgcaaccacaaccaactcccggcccttgctaacacctcggtccacgcagggatgatgggcgtgaattcgttcagtggacaactggccgtgaatgtctccgaggcatcacaagccaccaatcagggtttcacccgcaactcaagcagcgtatcaccacacgggtacgtgccgagcaccactccccagcagaccaactataactccgtcaccacgagcatgaacggatacggctctgccgcaatgtccaatttgggcggctcccccaccttcctcaacggctcagctgccaactccccctatgccatagtgccatccagccccaccatggcctcctccacaagcctcccctccaactgcagcagctcctcgggcatcttctccttctcaccagccaacatggtctcagccgtgaaacagaagagtgctttcgcaccagtcgtcagaccccagacctccccacctcccacctgcaccagcaccaacgggaacagcctgcaagcgatatctggcatgattgttcctcctatgtgaaagaattgccttgaagaattgtattaatgaagaggttggattctgctacagagagtaatctgatacaagtcccagagtggaacttttaactcaggcctttttaagaggaatcacaataactgcagatttttaaacaaacaaaatcaccgaccttgcaaatactgaaattggaagagggatctgcaagtgcagggtgttggttaaagttgtacctcccaagtatttgggggatatatttattctgtattgacaaaagcaaatccactttttctttttcttttttttttttaagcttaattctgcaatcatttgtcttttataaaccgtaaagctctatacaagggacactataaataagactccatgttttaatttatgatgtttttaaagctgtgtaaaggaagaatgaagtggtgatatttacaaaaaagtaaaaaaaaaaaaaaagaaaaaaaggaaaaaaaaaaaagcttgtatgggacagaataggaatgccagttagattttttagaaaaactaagggtcggcttttgcgccttaaagcatatcaaatggtagttagctcagacagtgcattttcaatatctaacttaacatgccaccccttagcagtgcaagcttatttatctcttttgtattgttgtcttaagcaactgtgtaaataaatgcagcctggaaagttaaaacgcgatgtaagtgatcacatttttccctctactcgaaaatccagtgcctctagacagattgttaaaactgcatatttaaatctgatctcattctctccttttacttaagtcagtttcttctgaagccgatggcctctcgagagcttggtggcacacatactgtgtgagaatctcctttgagacttcatggaacagggtccaggcacagaattccacactctgccctccagttaacaagccaaaacctcacgtacgctcccccatttgcaagtttaaagtttactcgtcctaaatgcagacttcatacctatcttccaaaagttgataaaagcaacgtggcaggtattttcgattttcccattccagatactgcctctatcagtcgacccttaccatttgtaacataatagattgaaacaacaggttaagtgctttggaattaagagttaaagggaaccgggggtggagaaaggaaaagaaggttgagacctccaaaacatagttttctgttctatgggaatttttgctctcattatctgggaagtgttcttaaaaataagaattaatagcaaagatgcagcaaagctctgaggatgcatttgcctgatatttttttctttgctcttgtgtttttgtatgtagttataaatactgtagattttttttttgtgattttttgccaaagttgttgttctatttatacattttaatgtcttaagacattttttcaatatcacaaaaagattttactgcgtattttgcaaagaaaaaaagctcactacctttagcttgcacatacttgcaaagttaattaaaaggctttttgttttaaaggggattttgtaaaatatccatataaataatgtatttatctttcgaatttgtacattgcttttcccttcttcctcttccttccacccccaatttattttattgtgtatgtttgctacgtgaaaagtgcgtatttgtttggtcacctacacttgtattagctgtttcaatgtgatttttaaacatttcatttatagttatttttagtattgttataaaccatgcttcagtttttttaatttccacccaaaagtcattgtctatttttgtattatttgtaagttaagaagttttttccaatatatggcaaaaaaaaaaaaatagtagcatattattcttgtagtatttagttcagtagatttaaaaaaaaatgtatcctttgctttggaagcttacaaaacaaccctaatgctgttttactctattaatatgcatggaatctctccctttggagtgacgcattttgtgcattaaattctagggagaaacttcatagaaatcaatgaacatactttctttcttaagtctgcttgtatatttcctctgtctttcacataaatataaaccagcagattggatgccttaacaatgcaaatcatattcatttcacttgtacattgtaactgtgcaccagaactgtcagtcatcactaacattctaagaaaaaagaaaaagaaaaaaagaaaaaaaaaaaaacaaagaaatcgaaaagcacaaaagaactgttttgttaccttaagacaatgtaactttttctagtagagcaagaaatcatttacaacaatgctgcaactgtgcatgcccccatatggattttgcaatggttttcactaggctgtcaagagtgcgatttttatgggttggggtgggtgggggaggggagttgtgggagggtaggggagggaggaaagctgtttttcatggtgagaaataataatgatgactaataataataaaaaaaactggaaaatgtaagcaaggtggacgcattgcttctcggtactcagaaaggttatctgaatttgcgtggtaagcgctggcctgaagatgtacacaagttaaagccatattttatctggtgagccccttaactgtttctgaaggaatgaacggtcagccgggaaggtgtccggcttagaccttgacaacagacactacccatctgtcagccatgtgcagtggttagaactcttcttgaaagtccaaagagcctttaaaatgtgtataatttgtgttttgttgcttctatttctattgattagatgaaaagacattctggccctctgatcctctttcttctccacaaaagttttacaaataaaagatgttcccctaaatacagaatatggctttaagaagaaaatgaaatgaagaattaaattaagatttcagtgttgggggaaaaaatacagcttctggatgactggattgcataacattgccctggcctcacattgtactaggatgcagcttaaatgaagtcatctctaaatctactaacctttcaccttcttatcaaaactttttgaatagacacacactgtacagttcaattgttagagaacctaactactgtagagattgttaaattttttttttttttttgcaaaaattcaagctgtaaaaacttttcaactttcacaatatttaattaaagttacttcctgtctgtga

### Immunofluorescence (IF) assay

First of all, we placed cells on coverslips and fixed them for 10 min by 4% paraformaldehyde. Then, cells were blockaded with 3% BSA and permeated with 0.5% Triton X-100. After that, the primary antibody (Abcam) against Ki67 (ab197234) was added for cultivation. Fluorescence-conjugated secondary antibody was supplemented for 2 h. DAPI was applied for staining nuclei. The staining situation was visualized by microscopy (Olympus). Bio-repeats run in triplicate.

### Colony formation

20,000–80,000 cells participated in the colony formation assay. After transfection, 500 cells were seeded in 6-well plates and were cultivated for 14 days. After that, we fixed cells by 4% paraformaldehyde and dyed cells with 0.1% crystal violet (Sigma-Aldrich). Eventually, the number of colonies was calculated manually. Bio-repeats run in triplicate.

### JC-1 assay

Firstly, we put 2.5 × 10^5^ cells in 96-well plates and conducted the centrifugation in cells for 5 min. Next, cells were loaded with JC-1 dye for 30 min after detaching the culture medium. Then, we utilized the fluorescent plate reader (Varioskan LUX; Thermo Fisher Scientific, Rockford, IL, USA) to detect the mitochondrial transmembrane potential (ΔΨm). Fluorescence microscopy (DMI8; Leica, Wetzlar, Germany) was utilized for observation. Bio-repeats run in triplicate.

### Transferase-mediated dUTP nick-end labeling (TUNEL) assay

First of all, 2.5 × 10^5^ transfected cells were fixed by 4% paraformaldehyde at room temperature in darkroom. Next, cells were permeated by 0.1% Triton X-100. After that, cells were rinsed with PBS for three times. On the basis of user guides, cell apoptosis was confirmed with One-Step TUNEL Apoptosis Assay Kit (Beyotime). Nucleus was stained by DAPI reagent. Fluorescence microscope was demanded for observation. Bio-repeats run in triplicate.

### Caspase-3/8/9 activity assay

Caspase-3/8/9 assay kit (Abcam, Cambridge, MA) was applied for measuring the activity of caspase-3/8/9 in cells. Then, 3 × 10^3^ cells were cultivated at room temperature for 72 h. After that, we plated cells in 6-well plates and analyzed them at 405 nm by microplate reader. This assay went through three independent repeats.

### Transwell assay

Transwell chamber (Corning Incorporated, Corning, NY, USA) was employed for assessing cell invasion. Then, 5 × 10^4^ cells were placed in the upper chamber with serum-free medium pre-coated with Matrigel gel (A3969002, BD Bioscience, San Jose, CA, USA) in the upper chamber. And the lower chamber was added with the complete medium (12558011). Then 24 h later, invaded cells were fixed and stained for visualization by the microscope (Olympus). Bio-repeats run in triplicate.

### Subcellular fractionation assay

In accordance with user guides, PARIS™ Kit (Invitrogen) was employed for separating cytoplasm and nucleus. Cells were lysed by cell fractionation buffer and then subjected to centrifugation. GAPDH and U6 served as controls. Bio-repeats run in triplicate.

### Fluorescence in situ hybridization (FISH)

The probes of FGD5-AS1 were obtained from RiboBio (Guangzhou, China) and labeled with Cy3 fluorescent dye. Cell nuclei were dyed by DAPI. Finally, the images were captured by fluorescence microscope (Olympus). This procedure was repeated for three times.

### Pull-down assay

Following the protocol, Pierce Magnetic RNA–Protein Pull-Down Kit (Thermo Fisher) was utilized to finish this assay. After obtaining protein extracts, we added magnetic beads for gathering the RNA–protein complex. Next, we eluted them and then analyzed via RT-qPCR. Bio-repeats run in triplicate.

### Luciferase reporter assay

Cells in the research were co-transfected with the pmirGLO vectors (Promega, Madison, WI, USA) containing the wild-type and mutant miR-124-3p binding sites to FGD5-AS1 fragment or G3BP2 3’-UTR and miR-124-3p mimics or NC mimics for 48 h. Luciferase Reporter Assay System (Promega) was hired to detect the relative luciferase activity. Bio-repeats run in triplicate.

### RNA immunoprecipitation (RIP)

Magna RIP™ RNA-Binding Protein Immunoprecipitation Kit (Millipore, Billerica, MA, USA) was used to carry out this experiment on the basis of user guide. We lysed cells which were in RIP lysis buffer and then treated them with magnetic beads conjugated with anti-Ago2 (TS-10X10ML-U, Millipore) or anti-IgG (MABE-253, Millipore). Finally, the retrieved RNA was assayed by RT-qPCR. This experiment went through three independent repeats.

### Bioinformatics methods

Related databases including miRmap, TargetScan, microT, miRanda and PicTar databases in starBase (http://starbase.sysu.edu.cn/index.php) were applied to select potential up miRNAs of G3BP2. UCSC (http://genome.ucsc.edu/) and PROMO (http://alggen.lsi.upc.es/cgi-bin/promo_v3/promo/promoinit.cgi?dirDB=TF_8.3/) were performed to select potential upstream transcription factor of FGD5-AS1. TCGA database (https://www.cancer.gov/about-nci/organization/ccg/research/structural-genomics/tcga) was applied to detect the expression pattern of FGA5-AS1 and EBF1 in sarcoma tissue samples.

### Statistical analysis

Each assay went through three independent repeats. Data were displayed as mean ± SD and analyzed via Student’s *t* test and one-way analysis of variance (ANOVA) by the utilization of GraphPad PRISM 6 (GraphPad, San Diego, CA, USA). Tukey and Dunnett were taken for the post hoc analysis. The significant difference was identified when *P* < 0 0.05.

## Results

### G3BP2 expression is up-regulated in osteosarcoma cells

**Fig. 1:**
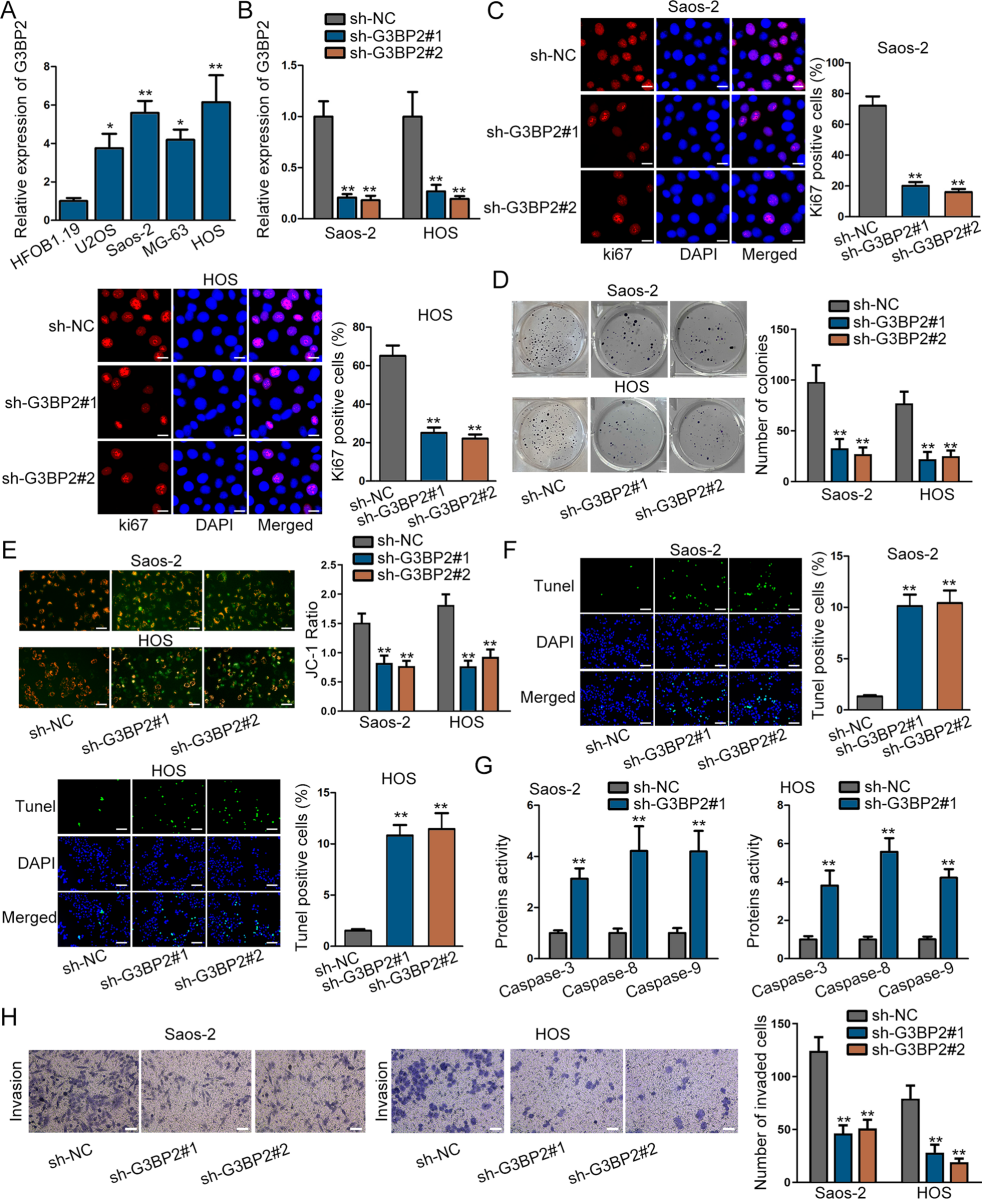
G3BP2 expression is up-regulated in osteosarcoma cells and facilitates the cell malignancy of osteosarcoma. **A** RT-qPCR of G3BP2 expression in osteosarcoma cell lines in contrast to normal osteoblast cell line. **B** RT-qPCR was taken to measure G3BP2 inhibition efficiency. **C**–**D** The proliferation of osteosarcoma cells after G3BP2 inhibition was analyzed by immunofluorescence (×20 magnified) as well as colony formation assays. **E**–**G** JC-1 (×100 magnified), TUNEL (×100 magnified) assay and caspase-3/8/9 assay were taken to evaluate cell apoptosis rate when G3BP2 was silenced. **H** Transwell assays (×100 magnified) measured cell invasion after G3BP2 was suppressed. ^*^P < 0.05, ^**^P < 0.01

For assessing the biological function of G3BP2, its expression status was first detected in osteosarcoma cell lines (Saos-2, U2OS, MG-63 and HOS) and normal cell line (HFOB1.19). Through RT-qPCR, we discovered the evidently up-regulation of G3BP2 in osteosarcoma cell lines in comparison with the normal cells **(**Fig. 1A). G3BP2 expression in Saos-2 and HOS cells displayed higher level, and thus, we silenced its expression in these two cells via transfecting sh-G3BP2#1/#2 (Fig. 1B). The percentage of Ki67 positive cells was significantly suppressed in cells transfected with sh-G3BP2 #1/2, as evidenced by IF assay **(**Fig. 1C). The repressive impact of G3BP2 silencing on osteosarcoma cell proliferation was further manifested by colony formation assay (Fig. 1D). As to cell apoptosis, JC-1, TUNEL as well as caspase-3/8/9 assays were demanded for detection. In JC-1 assay, we noticed that green fluorescence was more than red fluorescence in sh-G3BP2-transfectced cells (Fig. 1E). Meanwhile, the TUNEL positive cells were enhanced after G3BP2 was inhibited, as evidenced by TUNEL assay **(**Fig. 1F). Moreover, the protein activity of caspase-3/8/9 was up-regulated in transfected cells (Fig. 1G). These assays elucidated that cell apoptotic capability could be facilitated by G3BP2 depletion. In the end, transwell assay was employed for detecting cell invasion. The outcomes demonstrated that the number of invaded cells was notably cut down by silencing G3BP2 in cells, suggesting cell invasive capability was suppressed by knockdown of G3BP2 (Fig. 1H). In short, we discovered up-regulation of G3BP2 in osteosarcoma cells, and G3BP2 could accelerate cell proliferative and invasive capabilities.

### G3BP2 is targeted by miR-124-3p in osteosarcoma cells

**Fig. 2:**
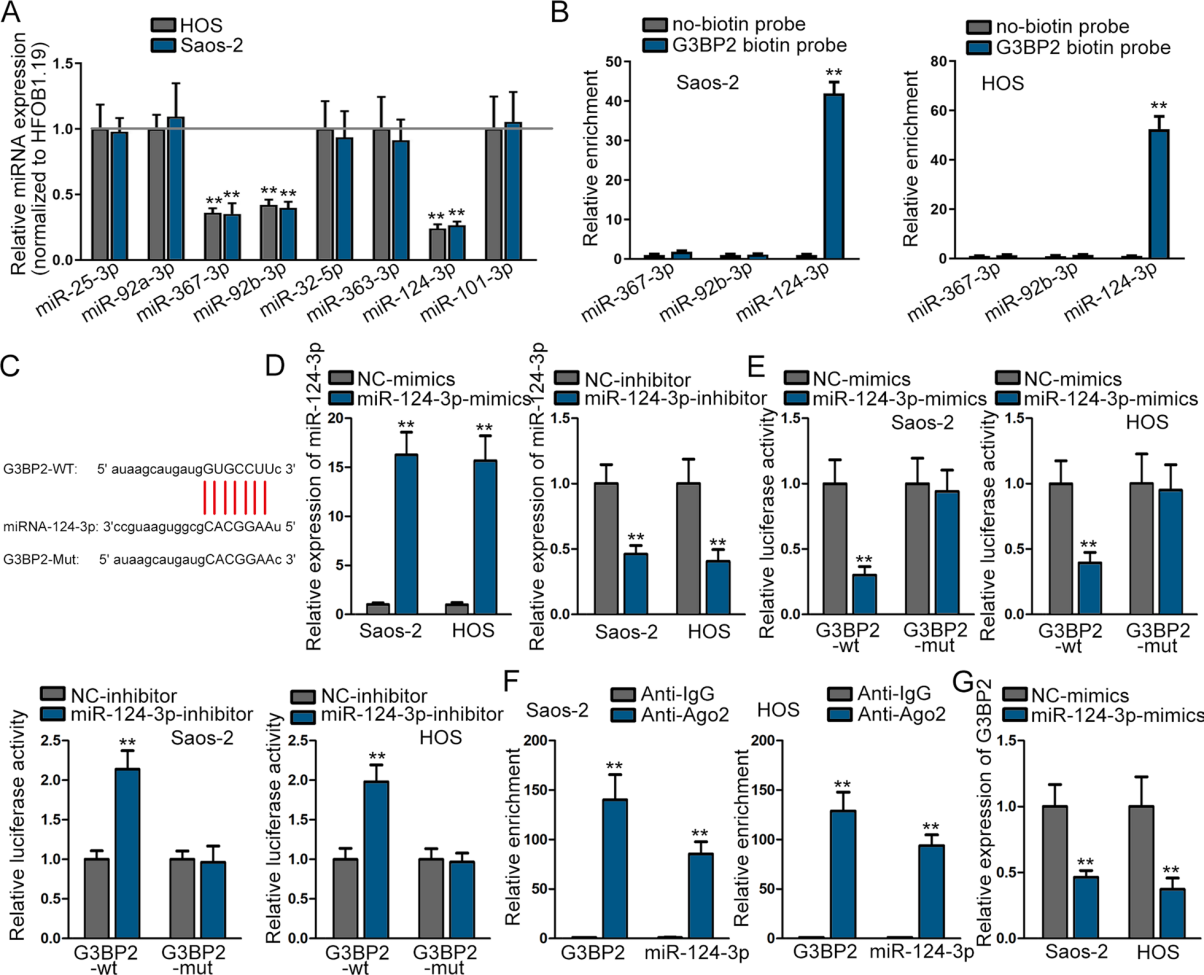
G3BP2 is the target of miR-124-3p in osteosarcoma cells. **A** The expression of the potential miRNAs in osteosarcoma cells was measured by RT-qPCR. **B** The enrichment of miR-124-3p, miR-92b-3p and miR-124-3p was measured in G3BP2 biotin probe. **C** The binding sites between G3BP2 and miR-124-3p were displayed via starBase. **D**–**E** Luciferase reporter assay was applied for verifying the combination of G3BP2 and miR-124-3p. **F** RIP assay detected whether G3BP2 and miR-124-3p can bind to Ago2 protein. **G** G3BP2 expression was measured after miR-124-3p overexpression via RT-qPCR. ^**^P < 0.01

MiRNAs have been confirmed to take part in the regulation of cancer process via negatively modulating mRNAs expression. Thus, we searched the upstream miRNA of G3BP2. According to the prediction of miRm1ap, TargetScan, microT, miRanda and PicTar database in starBase (http://starbase.sysu.edu.cn/index.php), nine miRNAs were found (Additional file 1: Figure S1A). Among all candidates, miR-367-3p, miR-92b-3p and miR-124-3p expressions were inhibited evidently in osteosarcoma cells (Fig. 2A). The outcomes of RNA pull-down assay then displayed that only miR-124-3p was abundantly enriched in the pull down of G3BP2 biotin probe, while the other miRNAs miR-367-3p and miR-92b-3p exhibited no changes (Fig. 2B). After that, we utilized starBase and figured out the binding sites of miR-124-3p and G3BP2 (Fig. 2C). We separately overexpressed and silenced miR-124-3p in osteosarcoma cells (Fig. 2D). It was then uncovered that the luciferase activity of G3BP2-WT could be prohibited by miR-124-3p mimics, but miR-124-3p silencing could render an opposite outcome. Inversely, the G3BP2-Mut group was almost unvaried (Fig. 2E). Furthermore, the result of RIP assay demonstrated that miR-124-3p and G3BP2 were both enriched in Ago2 antibody, suggesting that they co-existed in RISC complex (Fig. 2F). Finally, we confirmed through RT-qPCR data that enhanced miR-124-3p expression could reduce G3BP2 expression in osteosarcoma cells (Fig. 2G). In a word, miR-124-3p negatively regulated G3BP2 osteosarcoma cells.

### LncRNA FGA5-AS1 acts as a sponge of miR-124-3p

**Fig. 3:**
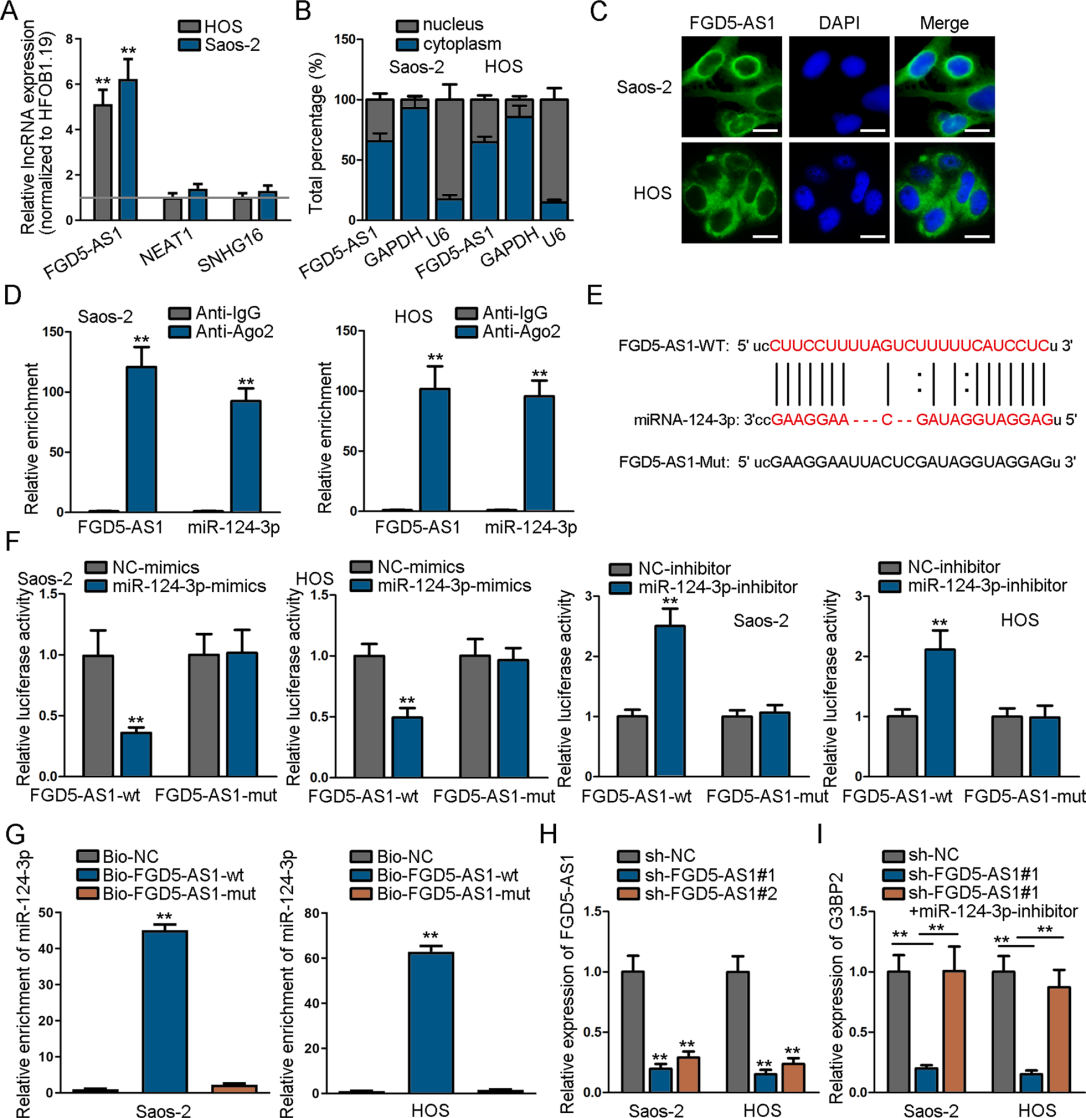
LncRNA FGA5-AS1 acts as a ceRNA to sponge miR-124-3p. **A** The expression of potential lncRNAs in HOS and Saos-2 cells normalized to HFOB1.19 cells. **B**–**C** The location of FGA5-AS1 in cells was identified through subcellular fractionation assay as well as FISH assay. **D** FGA5-AS1 and miR-124-3p enrichment in Ago2 group was measured via RIP assay. **E** The binding sites between FGA5-AS1 and miR-124-3p. **F** The combination of FGA5-AS1 and miR-124-3p was assessed via luciferase reporter assay. **G** MiR-124-3p enrichment in Bio-FGA5-AS1-WT/Mut groups was analyzed by RNA pull-down assay. **H** FGA5-AS1 expression was knocked down in osteosarcoma cells. **I** G3BP2 expression was assessed by RT-qPCR when FGA5-AS1 and miR-124-3p were suppressed in cells. ^**^P < 0.01

Competing endogenous RNA (ceRNA) pattern has been investigated in human cancers in recent years, and it is confirmed that lncRNA can sponge miRNAs via acting as a ceRNA to regulate mRNA expression, so as to inhibit or promote cancer progression. Thus, we tried to discover potential lncRNA that may modulate miR-124-3p through this way. Through utilizing starBase (selection condition: CLIP-Data >  = 5; pan-Cancer >  = 4), three lncRNAs (FGA5-AS1, NEAT1 and SNHG16) that could bind to miR-124-3p were predicted. As we could see through RT-qPCR, only FGA5-AS1 was expressed notably high in HOS and Saos-2 cells normalized to HFOB1.19 (Fig. 3A). Hence, we selected FGA5-AS1 to conduct following experiments. We also verified through TCGA database (https://www.cancer.gov/about-nci/organization/ccg/research/structural-genomics/tcga) that FGA5-AS1 exhibited up-regulated expression in sarcoma tissue samples (Additional file 1: Figure S1B).

Through subcellular fractionation assay as well as FISH assay, we confirmed that FGA5-AS1 was mainly distributed in cell cytoplasm, which signified that FGA5-AS1 had the possibility to act as ceRNA in cells at post-transcriptional level (Fig. 3B–C). Moreover, the result of RIP assay further suggested the enrichment of both FGA5-AS1 and miR-124-3p in Ago2 groups, elucidating that they were co-existed in RISC complex (Fig. 3D). Similarly, the binding sites between FGA5-AS1 and miR-124-3p were presented through starBase (Fig. 3E). Subsequently, it was shown that the luciferase activity of osteosarcoma cells was declined in FGA5-AS1-WT group transfected with miR-124-3p mimics, and an opposite result was seen after miR-124-3p was down-regulated (Fig. 3F). Besides, it was proven that miR-124-3p could be enriched in the pull down of biotinylated FGA5-AS1-WT, which further proved the combination between FGA5-AS1 and miR-124-3p (Fig. 3G). Consequently, we knocked down FGA5-AS1 expression in cells via transfecting sh-FGA5-AS1#1/#2 (Fig. 3H) and detected G3BP2 expression to evaluate their correlation. The results illustrated that G3BP2 expression could be inhibited by FGA5-AS1 depletion, while it was normalized after miR-124-3p inhibition (Fig. 3I). Taken together, lncRNA FGA5-AS1 sponged miR-124-3p via serving as a ceRNA to adjust G3BP2 expression in osteosarcoma cells.

### FGA5-AS1 accelerates osteosarcoma progression by up-regulating G3BP2

**Fig. 4:**
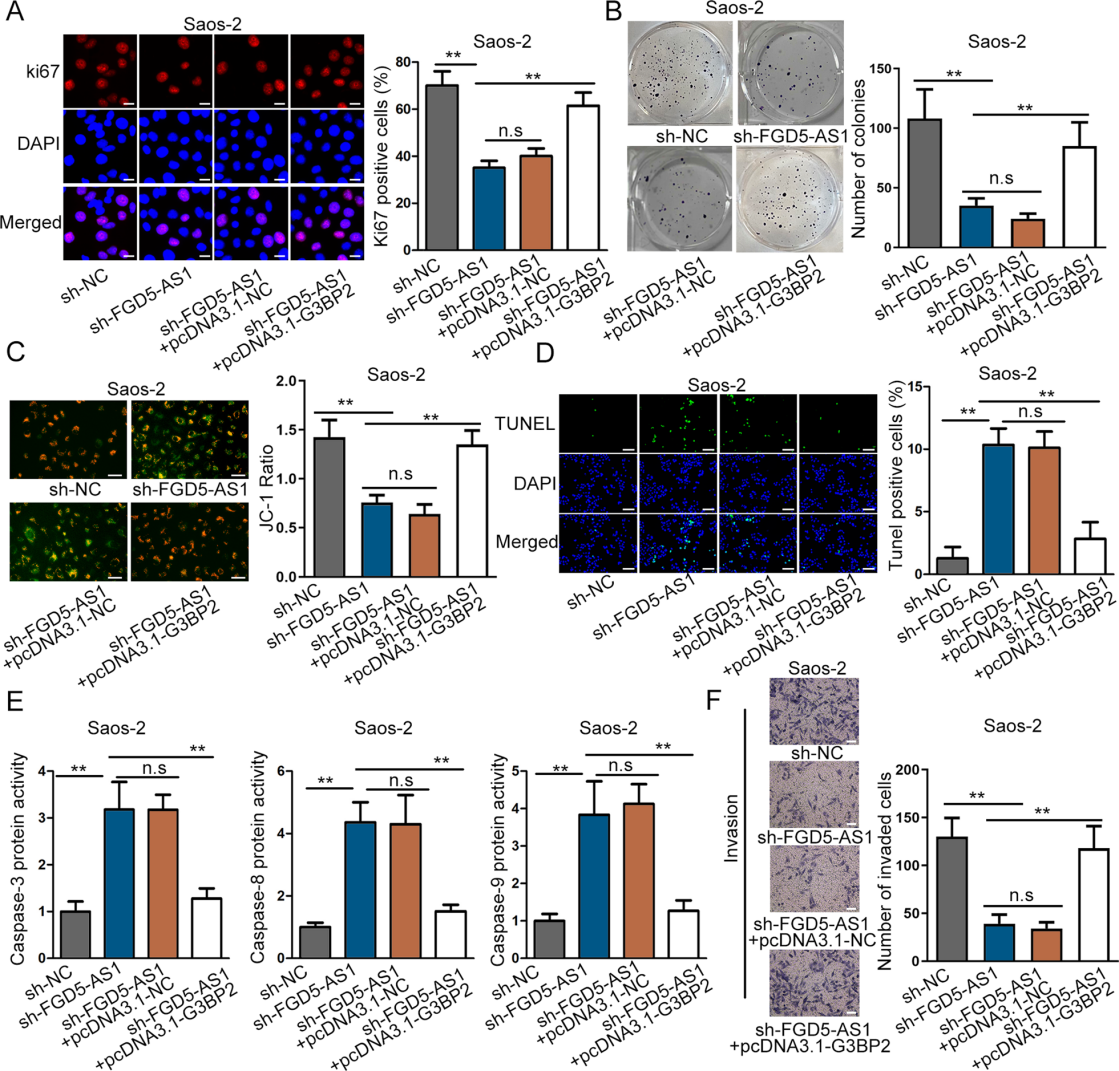
FGA5-AS1 accelerates osteosarcoma progression by up-regulating G3BP2. **A**–**B** Cell proliferation was tested by immunofluorescence and colony formation assays in different groups. **C**–**E** JC-1, along with TUNEL assay and caspase-3/8/9 assay was taken to evaluate cell apoptosis rate in different groups. **F** Transwell assays measured cell invasion under different conditions. ^**^P < 0.01, n.s.: no significance

For purpose of verifying the regulatory relationship between FGA5-AS1 and G3BP2, we implemented a chain of functional rescue assay. Through IF as well as colony formation assay, the cell proliferation was inhibited by FGA5-AS1 silencing, but this impact was offset by overexpressed G3BP2 expression (Fig. 4A–B, Additional file 2: Figure S2A–B). Following, cell apoptosis was confirmed by JC-1 assay, TUNEL staining and caspase-3/8/9 detection, and the outcomes suggested that down-regulation of FGA5-AS1 could enhance cell apoptosis ability, while co-transfection of pcDNA3.1-G3BP2 reversed the promoting effect (Fig. 4C–E, Additional file 2: Figure S2C–E). At last, we found through transwell assay that the repressive cell invasion caused by FGA5-AS1 down-regulation could be recovered by G3BP2 overexpression (Fig. 4F, Additional file 2: Figure S2F). Accordingly, we concluded that FGA5-AS1 accelerated osteosarcoma progression by up-regulating G3BP2.

### EBF1 activates FGA5-AS1 expression in osteosarcoma cells

**Fig. 5:**
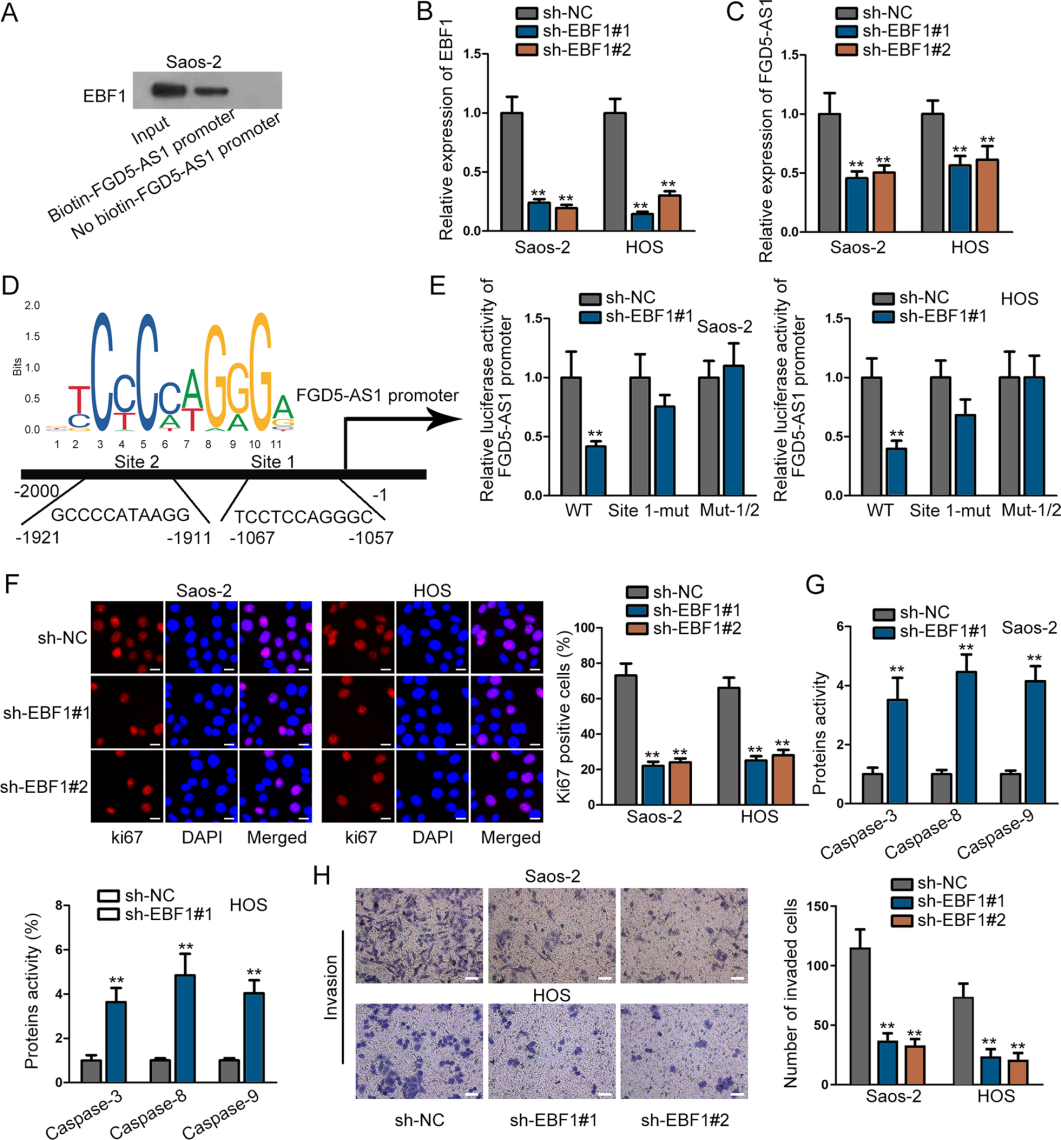
EBF1 activates FGA5-AS1 expression in osteosarcoma cells. **A** DNA pull-down assay detected the binding situation of EBF1 and FGA5-AS1 promoter. **B** EBF1 expression was inhibited in osteosarcoma cells. **C** FGA5-AS1 expression was tested via RT-qPCR when EBF1 was silenced. **D** The binding sites of FGA5-AS1 promoter and EBF1. **E** Luciferase reporter assay was performed to verify the binding sites of FGA5-AS1 promoter and EBF1. **F** The proliferation of osteosarcoma cells was assessed by immunofluorescence assay after EBF1 was prohibited. **G** Cell apoptosis upon EBF1 silencing was assessed by caspase-3/8/9 assay. **H** Cell invasion after EBF1 knockdown was verified by transwell assay. ^**^P < 0.01

The up-regulation of lncRNA expression has been confirmed to be closely associated with upstream transcription factor. Thus, we utilized to UCSC (http://genome.ucsc.edu/) and PROMO (http://alggen.lsi.upc.es/cgi-bin/promo_v3/promo/promoinit.cgi?dirDB=TF_8.3/) databases to look for the transcription factor of FGA5-AS1 (Additional file 3: Figure S3A). Accordingly, we found that EBF transcription factor 1 (EBF1) might be the transcription factor of FGA5-AS1. With the help of DNA pull-down assay, we confirmed that EBF1 can be pulled down by biotinylated FGA5-AS1 promoter, suggesting that EBF1 can bind to FGA5-AS1 promoter (Fig. 5A, Additional file 2: Figure S2G). In addition, it was verified through TCGA database that EBF1 was up-regulated in sarcoma tissue samples (Additional file 2: Figure S2H). Then, we suppressed EBF1 expression in cells via the sh-EBF1#1/#2 transfection (Fig. 5B) and detected its influence on FGA5-AS1 expression via RT-qPCR. FGA5-AS1 expression was verified to be reduced upon EBF1 silencing (Fig. 5C). Next, we utilized JASPAR (http://jaspar.genereg.net/) website and found two binding sites between FGA5-AS1 promoter and EBF1 (Fig. 5D). Moreover, luciferase reporter assay was performed and the results illustrated that the luciferase activity of wild-type FGA5-AS1 promoter could be inhibited by EBF1 depletion, while that of mutant FGA5-AS1 promoter was almost unchanged (Fig. 5E). Thus, these outcomes demonstrated that EBF1 could bind to FGA5-AS1 promoter.

Next, we detected the biological function of EBF1 on osteosarcoma cells. The result of IF assay indicated that the ratio of Ki67 positive cells was lessened by EBF1 inhibition (Fig. 5F). Then, the protein activity of caspase-3/8/9 was observed to be accelerated, suggesting that cell apoptosis could be enhanced by EBF1 inhibition (Fig. 5G). In the end, cell invasive ability was verified to be hampered by down-regulation of EBF1, as evidenced by transwell assay (Fig. 5H). In a word, EBF1 acts as an oncogene in osteosarcoma cells.

## Discussion

Osteosarcoma is the most frequent primary bone sarcomas, and the highest incidence is in children and adolescents, while a second smaller peak of incidence lies in elderly individuals over 60 years [1]. We aimed at unveiling the potential of G3BP2 as a biomarker for osteosarcoma treatment which has been reported to be with high expression in cancer cells such on prostate cancer [4] and BC [5]. During our investigations, we first displayed the high expression of G3BP2 in osteosarcoma cells through RT-qPCR data. Then, the results of loss-of-function assays proved that knockdown of G3BP2 in osteosarcoma cells resulted in the repression of cell proliferation and invasion. Accordingly, we got the conclusion that G3BP2 served as an oncogene in osteosarcoma, which was consistent with the previous findings we mentioned above.

Recently, increasing researches have indicated that lncRNAs can serve as ceRNA to regulate mRNA expression via sponging miRNA [18, 19]. The ceRNA pattern has been indicated to be pivotal in the process of cancer [20]. For example, lncRNA-CDC6 sponged miR-215 to target CDC6, thereby accelerating BC progression [21]. LncRNA FAM225A could expedite cell migration of nasopharyngeal carcinoma through playing as a ceRNA to regulate miR-590-3p/miR-1275/ITGB3 axis [22]. In our study, we firstly discovered the possible miRNA which could bind to G3BP2. Through the bioinformatics tools and mechanism assay, miR-124-3p was proved to target G3BP2 in osteosarcoma cells. Besides, a negative correlation between G3BP2 and miR-124-3p expression was revealed in osteosarcoma cells. As for miR-124-3p, it has been illustrated to suppress cell migration in gastric cancer via targeting ITGB3 [23]. Importantly, miR-124-3p was proved to exert the antitumor effect in osteosarcoma [24].

Then, for the purpose of deeply investigating the ceRNA network, we started to search for suitable lncRNA for miR-124-3p, and FGA5-AS1 was selected. For determining the possibility of FGA5-AS1 acting as a ceRNA, we detected its location in cells and discovered that it mainly distributed in cytoplasm. After verification of mechanism assay, miR-124-3p was verified to be sponged by FGA5-AS1 in osteosarcoma. Moreover, FGA5-AS1 was expressed evidently high in osteosarcoma cells and positively associated with G3BP2. Previously, FGA5-AS1 was proved to exert the carcinogenic effect in CRC [9], NSCLC [10] and oral cancer [11]. In rescue assays, it was indicated that overexpressed G3BP2 could reverse the repressive effect of inhibiting FGA5-AS1 on osteosarcoma process.

Transcription factors have been frequently identified in human cancers and activated the lncRNA expression which took part in regulating cancer malignant process (25, 26). After utilizing the bioinformatics tools, we distinguished that EBF1 may be the transcription factor of FGA5-AS1. Then, we carried out mechanism assay including DNA pull-down and luciferase reporter assay; the results verified the binding capacity between EBF1 and the FGA5-AS1 promoter.

In conclusion, we demonstrated through this research that G3BP2 was expressed obviously high in osteosarcoma cells, and FGA5-AS1 accelerated osteosarcoma cell proliferation and invasion by functioning as a ceRNA to sponge miR-124-3p to up-regulate G3BP2 expression, which might supply a new therapeutic target for osteosarcoma treatment. One of the limitations of our present study lies in the lack of in vivo as well as clinical manifestation of our findings, which are needed to be further implemented in the near future to further support our research data. Furthermore, we will make more efforts to collect related osteosarcoma tissue and patient samples to verify the correlation between G3BP2 and the clinical features of osteosarcoma patients.

## Supplementary Information

**Additional file 1. Figure S1** (A) The possible miRNAs were predicted by miRmap, TargetScan, microT, miRanda and PicTar database in starBase. (B) TCGA database was applied to detect the expression of FGA5-AS1 in sarcoma tissue samples compared with normal tissue samples. *P < 0.05

**Additional file 2. Figure S2** (A–B) The proliferation of HOS cell line in different groups was verified through immunofluorescence as well as colony formation assays. (C-E) JC-1, together with TUNEL assay and caspase-3/8/9 assay was performed to evaluate cell apoptosis rate. (F) Transwell assays were carried out to measure cell invasion. (G) The binding situation of EBF1 and FGA5-AS1 promoter in HOS cells was measured by DNA pull down assay. (H) TCGA database result of EBF1 expression in sarcoma tissue samples compared with normal tissue samples. *P < 0.05, **P < 0.01, n.s.: no significance

**Additional file 3. Figure S3** (A) UCSC and PROMO were taken to predict the upstream potential transcription factor of FGD5-AS1

## Data Availability

Not applicable.

## Abbreviations

G3BP2: G3BP stress granule assembly factor 2
FGD5-AS1: FGD5 antisense RNA 1
EBF1: EBF transcription factor 1
lncRNAs: Long noncoding RNAs
mRNA: Messenger RNA
miRNA: MicroRNA
ceRNA: Competing endogenous RNA
ATCC: American Type Culture Collection
FBS: Fetal bovine serum
qRT-PCR: Real-time quantitative polymerase chain reaction
DAPI: 4’,6-Diamidino-2-phenylindole
TUNEL: Terminal deoxynucleotidyl transferase (TdT) dUTP Nick-End Labeling
FISH: Fluorescence in situ hybridization
RIP: RNA immunoprecipitation
WT: Wild type
Mut: Mutant
SD: Standard deviation
ANOVA: Analysis of variance
