# Supplementary figures and images for: EBF1-mediated up-regulation of lncRNA FGD5-AS1 facilitates osteosarcoma progression by regulating miR-124-3p/G3BP2 axis as a ceRNA
Source: J Orthop Surg Res. 2022 Jun 27;17:332. doi: 10.1186/s13018-022-03181-7 (PMC9235248; doi:10.1186/s13018-022-03181-7)

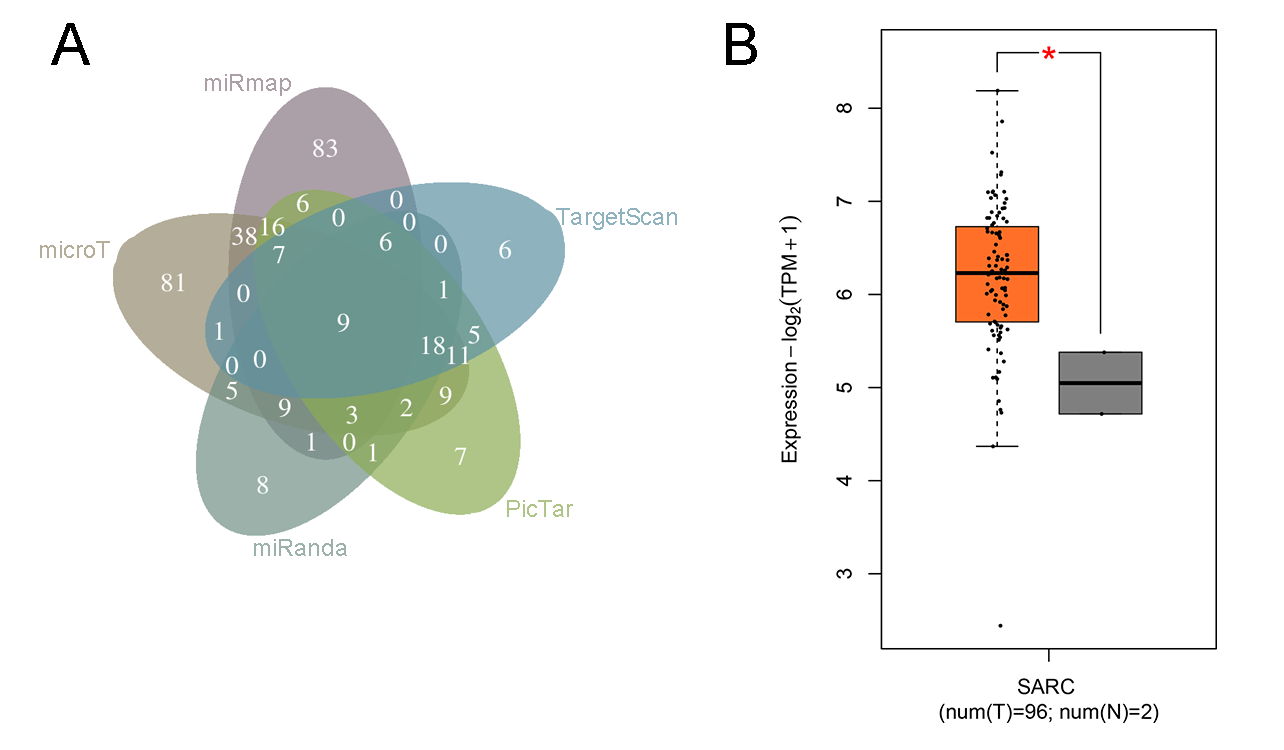

Supplement: Supplementary file 1 — Additional file 1. Figure S1 (A) The possible miRNAs were predicted by miRmap, TargetScan, microT, miRanda and PicTar database in starBase. (B) TCGA database was applied to detect the expression of FGA5-AS1 in sarcoma tissue samples compared with normal tissue samples. *P < 0.05 [file 13018_2022_3181_MOESM1_ESM.tif]

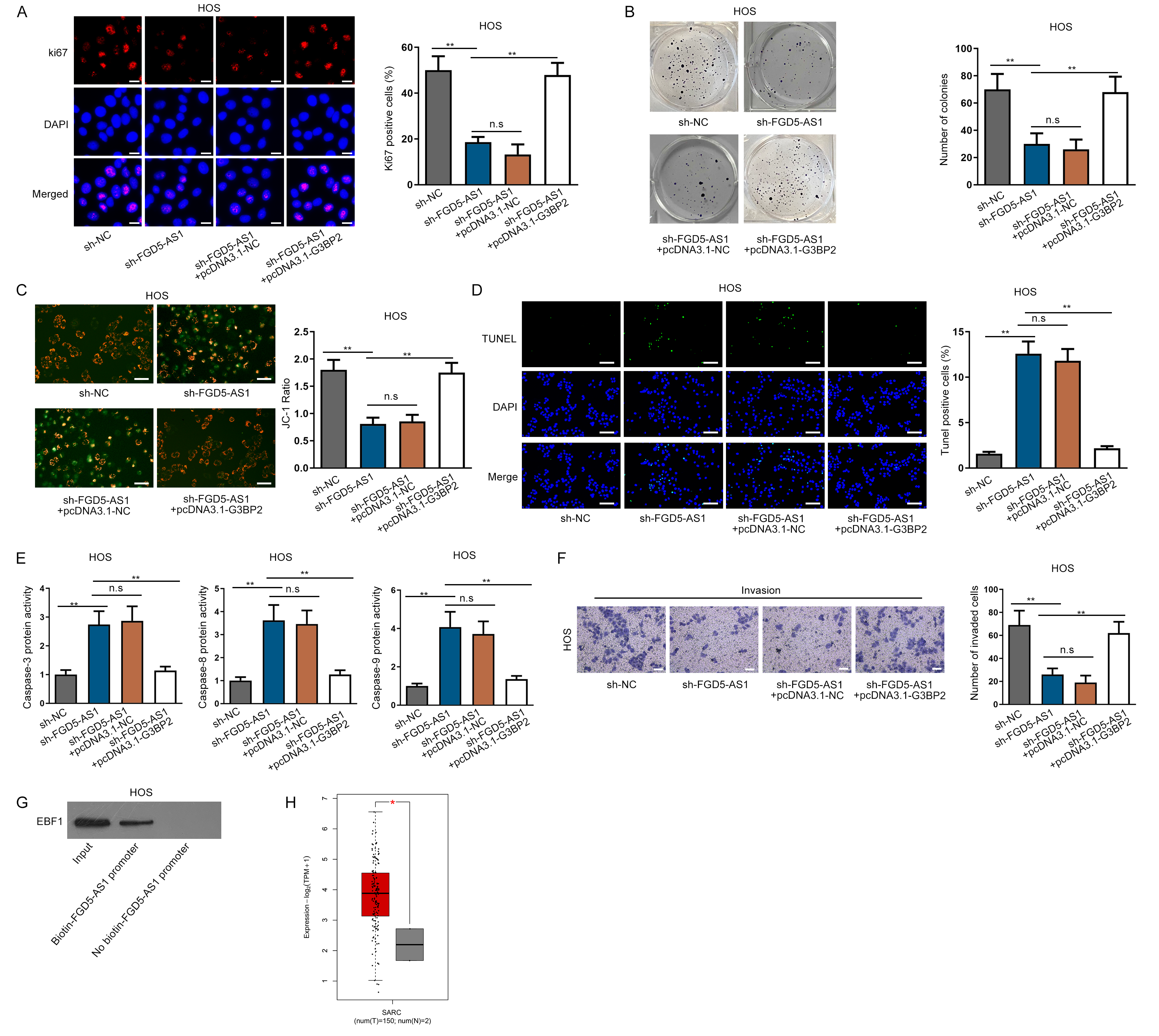

Supplement: Supplementary file 2 — Additional file 2. Figure S2 (A–B) The proliferation of HOS cell line in different groups was verified through immunofluorescence as well as colony formation assays. (C-E) JC-1, together with TUNEL assay and caspase-3/8/9 assay was performed to evaluate cell apoptosis rate. (F) Transwell assays were carried out to measure cell invasion. (G) The binding situation of EBF1 and FGA5-AS1 promoter in HOS cells was measured by DNA pull down assay. (H) TCGA database result of EBF1 expression in sarcoma tissue samples compared with normal tissue samples. *P < 0.05, **P < 0.01, n.s.: no significance [file 13018_2022_3181_MOESM2_ESM.tif]

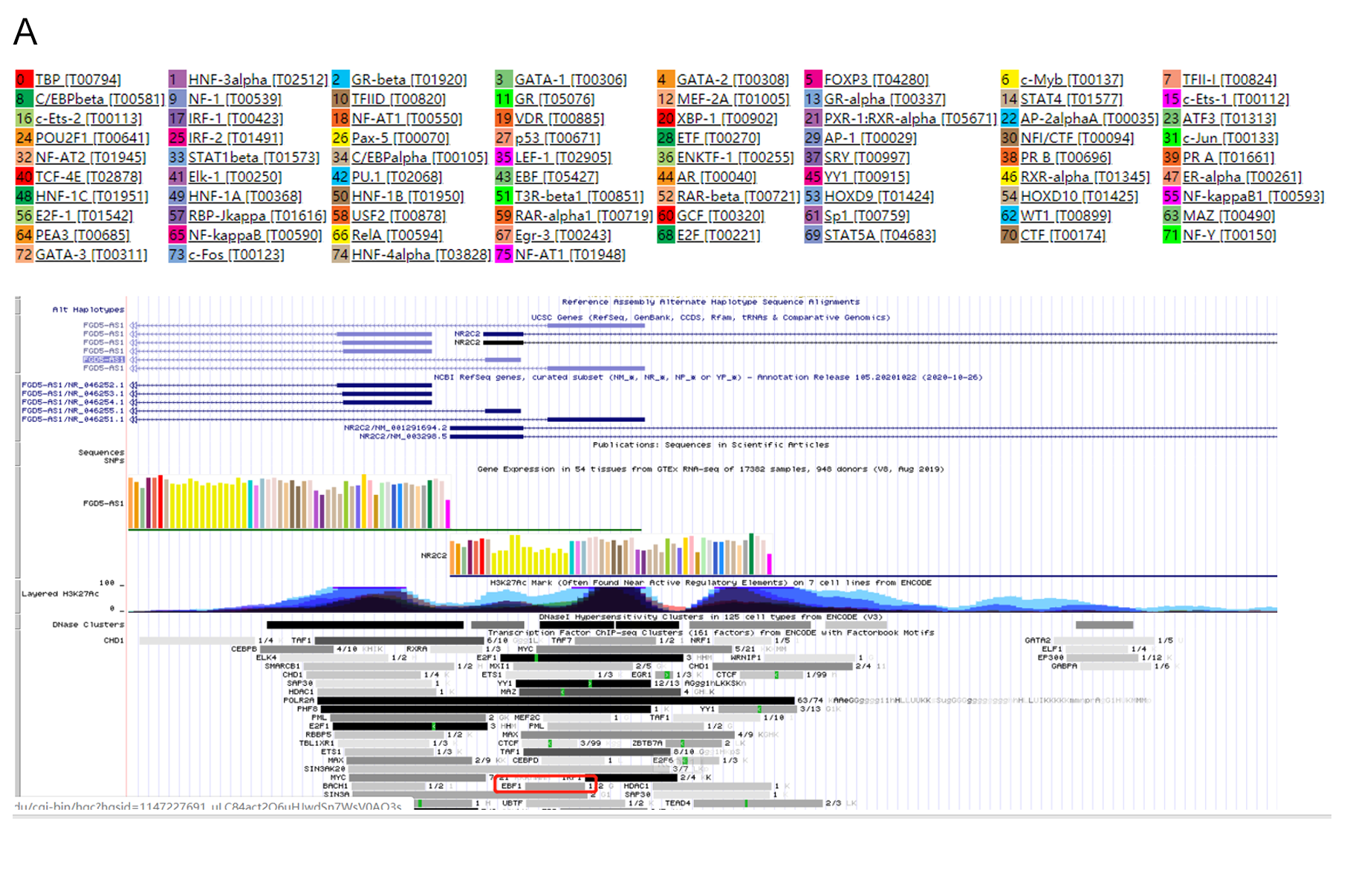

Supplement: Supplementary file 3 — Additional file 3. Figure S3 (A) UCSC and PROMO were taken to predict the upstream potential transcription factor of FGD5-AS1 [file 13018_2022_3181_MOESM3_ESM.tif]
